# The utility of point-of-care urinary lipoarabinomannan testing for the diagnosis of tuberculosis in critically ill patients: a prospective observational study

**DOI:** 10.1186/s12879-021-05979-y

**Published:** 2021-03-19

**Authors:** Kim de Vasconcellos, Praksha Ramjathan, Dhivendra Singh

**Affiliations:** 1 Department of Critical Care, King Edward VIII Hospital, Durban, South Africa; 2 Discipline of Anaesthesiology and Critical Care, School of Clinical Medicine, University of KwaZulu-Natal, Durban, South Africa; 3 Department of Medical Microbiology, School of Laboratory Medicine and Medical Science, University of KwaZulu-Natal and National Health Laboratory Services, King Edward VIII Hospital, Durban, South Africa

**Keywords:** Urinary lipoarabinomannan, Point-of-care, tuberculosis, Critically ill

## Abstract

**Background:**

Tuberculosis is a major global public health concern. Patients with tuberculosis who require critical care have a high mortality and delay in initiating antituberculous therapy is associated with increased mortality. Lipoarabinomannan (LAM) is a lipopolysaccharide found in the cell wall of *Mycobacterium tuberculosis*. Urinary LAM may be used as a bedside diagnostic test for tuberculosis.

**Methods:**

The study was a single centre, prospective observational study that compared the utility of urinary LAM with conventional tuberculosis diagnostic modalities in patients with suspected tuberculosis who required intensive care admission. Urinary LAM testing was performed using the Alere Determine TB LAM Ag lateral flow assay test strips. A patient was classified as having confirmed tuberculosis if they met the following criteria: a clinical presentation compatible with tuberculosis, with either a positive TB culture, a positive GeneXpert, or a histological diagnosis of tuberculosis.

**Results:**

Fifty patients were included in the study, with 12 having confirmed tuberculosis. All patients received mechanical ventilation, and the ICU mortality was 60%. Urinary LAM had a sensitivity of 50.0% (95% CI, 21.1 to 78.9%) and a specificity of 84.2% (95% CI, 68.8 to 94.0%) for confirmed tuberculosis.

**Conclusion:**

Urinary LAM allows for rapid bedside diagnosis of tuberculosis in critically ill patients. A positive urinary LAM should prompt consideration to initiate antituberculous treatment while the results of further diagnostic testing are awaited.

## Background

Tuberculosis (TB) is a major contributor to mortality globally [1]. The mortality rate of TB which necessitates intensive care unit (ICU) admission ranges between 15.5 and 65.9% [2–4]. The gold standard for TB diagnosis is culture of pulmonary or extrapulmonary specimens. Culture can take up to 6 weeks and more rapid tests are needed for critically ill patients in an intensive care setting. The GeneXpert is an automated platform that offers a more rapid test but still requires sophisticated equipment and a laboratory to process the specimen. GeneXpert has the greatest utility for diagnosis of pulmonary TB and may have lower sensitivity or may not be technically feasible for diagnosis of extrapulmonary TB. The sensitivity of GeneXpert is lower, and the risk of extrapulmonary TB is higher, in HIV positive patients [5–7]. A diagnostic test for TB would ideally be able to detect pulmonary and extrapulmonary TB and perform well in both HIV positive and negative patients. Delays in diagnosis and initiation of treatment result in higher mortality rates [2, 3]. The ideal test would thus also be point of care and would deliver an answer at the bedside.

Urine antigen testing is an attractive option for diagnosis of TB as it is an easily obtained specimen and does not generate dangerous aerosols [8]. Many mycobacterial antigens can be detected in urine, of which the most promising has been the cell wall lipopolysaccharide lipoarabinomannan (LAM) [9, 10]. LAM is one of the lipopolysaccharides that make up the mycobacterial cell wall. The precise mechanism by which LAM gets into the urine is unknown [5]. The Alere Determine TB LAM Ag test is a simple, easy to use lateral flow assay. The advantage of the TB LAM Ag test is that it can be done at the patient’s bedside and results are available immediately. Furthermore, patients with extrapulmonary TB may have negative sputum and endotracheal aspirates using GeneXpert but may have LAM antigenuria which the TB LAM Ag test could detect [8]. The reported sensitivity and specificity of TB LAM varies according to the cohort studied: in particular whether the patients had TB symptoms or were an unselected cohort; whether the participants were outpatients or were hospitalised; and according to CD4 lymphocyte count [11]. In a recent Cochrane review of the performance of TB LAM in HIV positive patients, Bjerrum et al. reported pooled sensitivities of 42% in patients with TB symptoms as opposed to 35% in patients who had not been assessed for features of TB; with corresponding specificities of 91 and 95% respectively. Pooled sensitivities were 29% in outpatients with TB symptoms and 52% in hospitalised patients with TB symptoms. The results for unselected patients were 31 and 62% respectively. Specificity varied from 96% in outpatients with TB symptoms to 87% in inpatients with symptoms, with the corresponding results for unselected patients being 95 and 84% respectively [11]. When symptomatic patients were stratified by CD4 count, the following results were noted: in patients with CD4 counts of over 200 cells/ul, sensitivity ranged from 9 to 27% and specificity from 83 to 99%; while in patients with CD4 counts of ≤100 cells/ul the corresponding results were 30–65% and 75–94% [11]. For patients with a CD4 lymphocyte count less than 50 cells/uL, Lawn and Peter reported sensitivities of 66.7 and 63.7%, which is comparable to a single Xpert MTB Rif test in that patient group [12, 13]. Most studies on urinary LAM have been restricted to HIV positive patients due to poor performance in HIV negative patients. A metanalysis by Minion et al., reported pooled sensitivities of 18% in HIV negative patients, as opposed to 56% in HIV positive patients [14].

No studies have been done using this test in the intensive care setting. Although previous studies may have included critically ill patients, findings for these patients have not been reported specifically. Furthermore, as the majority of studies required informed consent from the participants, it is likely that most ICU patients were excluded as they would not have been able to provide consent due to their severity of illness and use of sedative drugs [13, 15, 16]. Given the high mortality associated with TB in the ICU, and given the association between delayed treatment of TB and increased mortality, we postulated that a rapid point of care test for TB would have potential clinical utility in the ICU [2, 3]. The TB LAM Ag test offers the potential for rapid bedside diagnosis of TB in ICU patients; however, its diagnostic performance varies between patient populations and as such it is unclear whether it would be appropriate for use in the intensive care unit. We hypothesised that due to their severity of illness, patients with TB who required ICU would be more likely to have TB LAM antigenuria, with this being less dependent on HIV status and CD4 count. This study thus attempted to evaluate whether the TB LAM Ag test is a useful adjuvant test in the intensive care setting by evaluating its diagnostic accuracy and potential clinical utility in both HIV positive and HIV negative patients admitted to ICUs with suspected tuberculosis.

## Methods

This was a prospective observational study of patients admitted to King Edward VIII Hospital Intensive Care Unit from 30/01/2018 to 06/01/2020. The study ICU is a closed, multidisciplinary ICU in a tertiary academic hospital in KwaZulu-Natal South Africa.

The following bodies provided permission for the study to proceed: The Biomedical Research Committee of the University of KwaZulu-Natal (BE 516/17), The Health Research Committee of the KwaZulu-Natal Provincial Department of Health and King Edward VIII Hospital. The study also complied with requirements for research of the National Health Laboratory Service (NHLS) who performed the relevant laboratory testing. As the target population consisted of critically ill ICU patients informed consent was waived. The study was thus conducted according to locally accepted ethical guidelines for research involving critically ill patients who are incapable of giving informed consent: the study should be of minimal risk, involve no alteration in the standard of care, be non-interventional and should be justified, of social value and in the interest of public health. As tuberculosis is a significant burden of disease in our setting the study was of social value and in the interest of public health. Furthermore, the risks of the study were minimised as all diagnostic procedures were conducted as part of routine patient care and were performed by medical and nursing staff experienced in treating critically ill patients. Interpretation of the LAM assay by the investigators did not influence the choice of antibiotics patients were on as part of the ICU treatment protocol and treating physicians were advised to follow accepted investigation and treatment algorithms. HIV status and CD4 counts did not influence the methods of the study in any way and the results remained confidential. Participant names, hospital numbers and HIV status were deidentified and stored electronically according to local guidelines. All data will be stored and disposed of after the required period. Funding was applied for and granted by the Critical Care Society of Southern Africa and the funding body had no input into any aspect of the study design or presentation of the study results.

All adult (age ≥ 18 years) patients admitted to the study ICU, who were investigated for TB as part of their routine clinical management were eligible for inclusion in the study. This included patients with a clinical suspicion of TB as either their primary or secondary diagnosis, as determined by their primary treating ICU physician. Patients who had been started on TB treatment by the referring clinicians were eligible for inclusion, and patients were eligible for inclusion irrespective of HIV status. Patients were excluded if they were < 18 years old, were anuric or did not have appropriate TB diagnostic testing or LAM testing performed.

Due to lack of previous data from a critical care setting, formal sample size calculations were deemed inappropriate. A pragmatic sample size of a minimum of 50, and a maximum of 100 patients over a 6-month period was chosen as this was deemed feasible in terms of time and resource constraints. Due to lower-than-expected admission numbers, the study recruitment period was extended (with ethical approval) until a minimum of 50 consecutive patients that met the study criteria were recruited.

All data (other than the LAM results) were obtained from the patients’ physical or electronic records and were obtained as part of their routine care. Data collected included demographic data, TB diagnostic data (GeneXpert, auramine, TB culture, histology, and fluid microscopy and chemistry from appropriate specimens and radiology results), HIV status and CD4 count, urine microscopy and culture and urinary TB LAM. TB diagnostic tests were limited to those requested by the treating physician, based on national testing guidelines, and were not standardised or adjusted for the purposes of this study. Urinary LAM testing was conducted using the Alere Determine TB LAM Ag lateral flow assay test strips (Abbott Laboratories, Lake Bluff, USA) as soon as logistically possible after a diagnosis of TB was considered, and appropriate diagnostic tests were ordered. Testing was performed on the first available fresh catheter urine specimen obtained during routine patient care. Testing was performed at the patient’s bedside using the recommended test procedure by a suitably trained member of the ICU staff, other than the patient’s primary physician. The tests were read and interpreted according to the manufacturer’s instructions, using the manufacturer’s reference card to first determine if a test was valid, and then to determine if the test result was positive or negative. A test was deemed valid if a visible line was noted in the “control” area of the test strip and was deemed positive if a visible line was noted in the “patient” area of the test strip that matched the manufacturer’s reference card for a positive result. A test without a valid control line was repeated and a result that did not meet the criteria for a positive result was deemed as negative. All results were confirmed by a second reviewer. Staff performing the LAM testing were not blinded to the patient’s clinical information but were unaware of the results of the TB diagnostic testing ordered at the time LAM was performed. The primary physician was not blinded to the LAM results but was advised that all treatment decisions should only be based on currently accepted treatment guidelines. GeneXpert MTB/RIF Ultra (Cepheid, Sunnyvale, CA USA) was performed according to the manufacturer’s instructions. TB culture was performed according to the standard laboratory procedure using the BACTEC mycobacteria growth indication tubes 960 system [BACTEC MGIT Becton Dickinson, USA].

The following definitions, modified from a previous study, were used to classify TB diagnostic category [17]:

- Confirmed TB: a clinical presentation compatible with TB, with a positive TB culture, or 1 positive GeneXpert, or a histological diagnosis of TB.
- Probable TB: a clinical-radiological picture highly suggestive of TB and TB treatment provided by attending physicians but without any of the above positive laboratory findings.
- No TB: no evidence of TB based on a negative TB culture and GeneXpert (or either test if only 1 testing modality performed), no radiological evidence of TB and a clinical decision not to provide/continue TB treatment.

The classification of patients into diagnostic categories was performed by an investigator blinded to the LAM results. The choice of reference standards and composite diagnostic categories was influenced by TB diagnostic guidelines current at the time of the study, the pragmatic nature of the study (TB diagnostic testing was at the discretion of the treating physician and was not standardised/modified for the purposes of the study), and the limited sensitivity of the potential reference standards when used in isolation.

All data was collected in Microsoft Excel and was analysed using IBM SPSS Statistics for Windows, Version 26.0 and MedCalc (MedCalc Software, Ostend, Belgium) diagnostic test evaluation calculator. Categorical variables were described as numbers and percentages and compared using the Chi-square test or Fisher’s exact test, where appropriate. Continuous data were described using median and interquartile range (IQR) as the distribution of these variables was non-Gaussian. These data were compared using the Mann-Whitney U-test. The diagnostic performance of TB LAM for both “confirmed” and “possible” TB was evaluated in terms of sensitivity, specificity, positive predictive value, negative predictive value, positive likelihood ratio, negative likelihood ratio, number needed to diagnose, and diagnostic accuracy [overall probability that a patient is correctly classified = sensitivity × prevalence + specificity × (1 − prevalence)], with 95% confidence interval (CI) where appropriate.

The study has been reported according to the Standards for Reporting Diagnostic Accuracy (STARD) guidelines of 2015 [18].

## Results

**Fig. 1:**
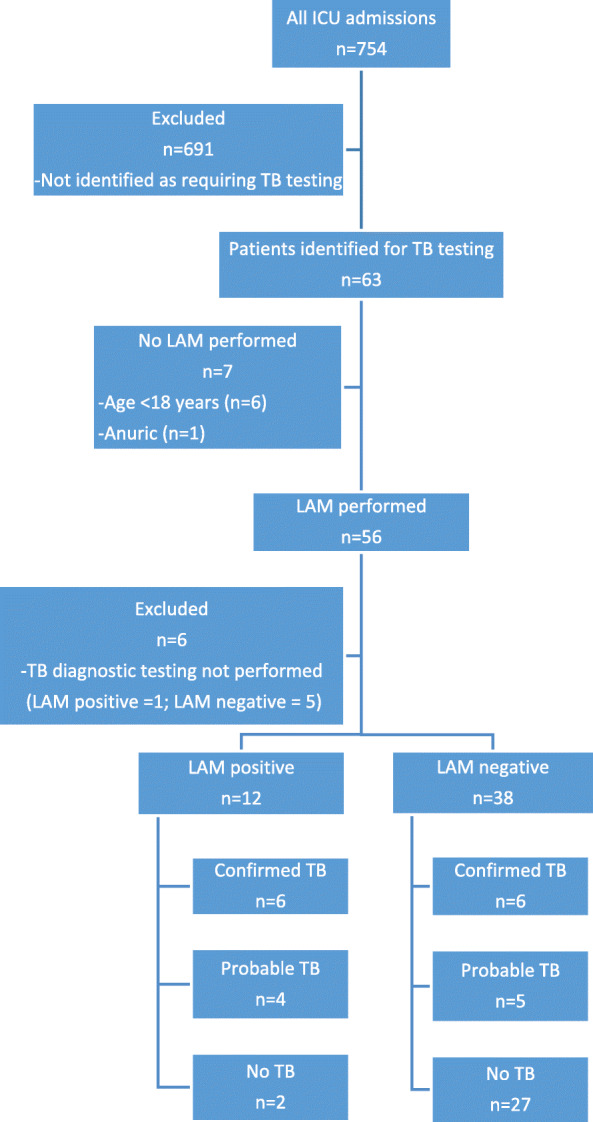
Flow diagram of study participants, LAM results and TB diagnostic categories

A total of 63 patients were screened for inclusion. Six were excluded as they were less than 18 years old, 1 for anuria, and 7 because none of the tests required to diagnose “confirmed TB” (GeneXpert, TB culture or histology results) were available despite planned testing. The reasons for the latter included inability to obtain an endotracheal aspirate or other appropriate specimen, death prior to sampling and clerical error. In the patient who was anuric and a urinary LAM specimen was not possible, the GeneXpert was positive. Of the 6 patients where the appropriate TB diagnostic test was not performed, 1 LAM result was positive. In total 50 patients were included in the study. Patient flow is shown in Fig. 1.

Table 1 highlights baseline demographic data, and ICU outcome and organ support data for the included patients. A total of 37 (74%) patients were investigated for TB as part of the work-up for a primary diagnosis of community-acquired pneumonia, 7 (14%) for disseminated TB, and 6 (12%) for community-acquired pneumonia as a secondary diagnosis.

**Table 1:** Demographic, organ support and outcome data for cohort

		*Median (IQR) or n (%)*
Age (years)		39 (30–53)
Gender	Female	29 (58%)
	Male	21 (42%)
HIV status	Positive	29 (58%)
	Negative	18 (36%)
	Unknown	3 (6%)
Mechanical ventilation		50 (100%)
Inotropic support		41 (82%)
Renal replacement therapy		12 (24%)
ICU length of stay (days)		7 (4–10)
ICU Outcome	Survived	20 (40%)
	Died	30 (60%)

A total of 12 (24%) patients had confirmed TB, with an additional 9 (18%) having probable TB (Fig. 1). All cases of confirmed or probable TB were deemed to have pulmonary or disseminated TB on clinical assessment. No cases were deemed to have isolated extrapulmonary TB. The results of TB diagnostic testing are shown in Table 2.

**Table 2:** Results of TB laboratory testing

	Total	Negative	Positive
*Per patient*^*a*^			
**LAM**	50	38 (76%)	12 (24%)
**GeneXpert**	49	41 (84%)	8 (16%)
**TB culture**	43	40 (93%)	3 (7%)
**Auramine**	12	7 (58%)	5 (42%)
**Histology**	2	0 (0%)	2 (100%)
*Per sample*^*b*^			
**GeneXpert**	69	59 (86%)	10 (14%)
**TB culture**	60	57 (95%)	3 (5%)

^*a*^ *The results in this category are aggregated results for each patient. A test may have been repeated multiple times per patient and in the case of repeat testing with discordant results, the test category would be reported as positive if there was a single positive result*

^*b*^ *The results in this category represent the results of each individual test and are not presented per patient*

**Fig. 2:**
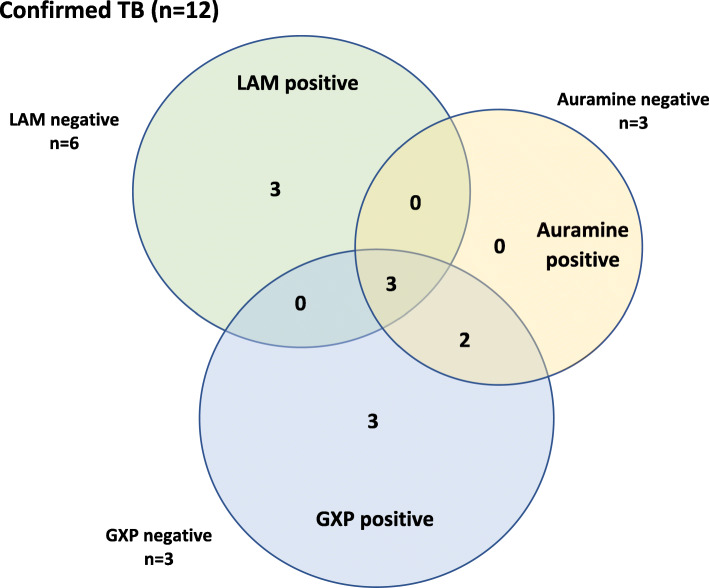
Results of rapid diagnostic tests for confirmed cases of TB. *Notes: Classification as confirmed TB required a clinical presentation compatible with TB, with a positive TB culture, a positive GeneXpert, or a histological diagnosis of TB. At least one rapid diagnostic test was positive in 11 patients with confirmed TB, while in one patient with a positive TB culture, rapid tests were negative. GXP = GeneXpert*

**Fig. 3:**
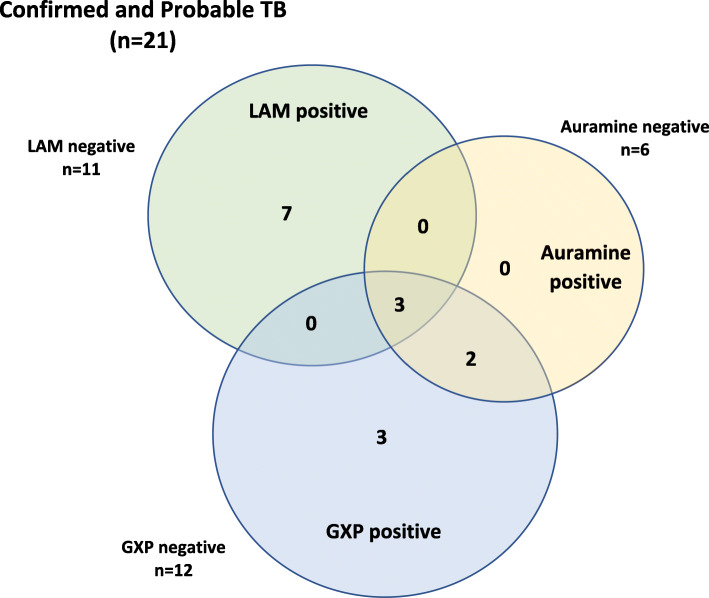
Results of rapid diagnostic tests for confirmed and probable cases of TB. *Notes: Classification as probable TB required a clinical-radiological picture highly suggestive of TB, with TB treatment provided by attending physicians but without positive TB culture, GeneXpert or histology. Of the 9 patients with probable TB, 4 had a positive TB LAM, while 5 had negative rapid diagnostic tests. GXP = GeneXpert*

The diagnostic performance of LAM for confirmed TB and for the composite outcome of confirmed and probable TB is shown in Table 3. GeneXpert was not performed in 1 patient with confirmed TB. GeneXpert thus detected 8 of 11 patients with confirmed TB in whom the test was performed (72.7% of confirmed TB, 95% CI 39.0–94.0%) and 8 of the 20 patients with the composite outcome of “confirmed” or “probable” TB (40.0% of composite outcome, 95% CI 19.1 to 64.0%). Of the 6 patients with confirmed TB who were LAM positive, 2 had a negative GeneXpert, and were diagnosed on TB culture, while 1 patient did not have a GeneXpert performed and TB was diagnosed on histology. Of the 3 patients with a positive TB culture, 2 (66.7%) had a positive LAM, while the GeneXpert was negative in all 3. The results of rapid diagnostic testing for patients with confirmed TB are shown in Fig. 2 and those for probable TB in Fig. 3.

**Table 3:** Performance of LAM for diagnosing confirmed TB and the composite outcome of confirmed and probable TB

	Confirmed TB	Confirmed + Probable TB
	*Value (95% CI)*	*Value (95% CI)*
**Sensitivity**	50.0% (21.1 to 78.9%)	47.6% (25.7 to 70.2%)
**Specificity**	84.2% (68.8 to 94.0%)	93.1% (77.2 to 99.2%)
**Positive Likelihood Ratio**	3.2 (1.3 to 8.0)	6.9 (1.7 to 28.3)
**Negative Likelihood Ratio**	0.6 (0.3 to 1.1)	0.6 (0.4 to 0.9)
**Positive Predictive Value**	50.0% (28.4 to 71.7%)	83.3% (55.0 to 95.4%)
**Negative Predictive Value**	84.2% (74.9 to 90.5%)	71.1% (61.7 to 78.9%)
**Accuracy**	76.0% (61.8 to 86.9%)	74.0% (59.7 to 85.4%)

The CD4 count was known in 26 of the 29 HIV positive patients. The median CD4 count was 156 cells/uL (IQR, 47–275 cells/uL). The median CD4 count in LAM negative patients was 124 cells/uL (IQR, 39–252 cells/uL), and was 374 cells/uL (IQR, 153–618 cells/uL) in LAM positive patients, *p* = 0.083. There was no statistically significant difference in the proportion of positive LAM tests according to HIV status or CD4 count. TB LAM was positive in 8 of 29 (27.6%) HIV positive patients and 4 of 18 (22.2%) HIV negative patients, *p* = 0.744. For patients with a CD4 count < 200 cells/uL, 2/15 (13.3%) were LAM positive, as opposed to 4/11 (36.4%) for those with a CD4 count ≥ to 200 cells/uL, *p* = 0.348. The corresponding figures for a CD4 count of < or ≥ to 100 cells/uL were 1/8 (11.1%) and 5/17 (29.4%) respectively, *p* = 0.380. Of the 2 patients classified as having “no” TB who had a positive LAM, one was HIV positive and one was HIV negative.

The ICU mortality rate in patients with no TB was 41.4% (12 of 29 patients) and was 85.7% (18 of 21) in patients with confirmed or probable TB, *p* = 0.002. In patients with a negative GeneXpert the mortality rate was 51.2% (21 of 41) as opposed to 100% in those with a positive GeneXpert, *p* = 0.010. For LAM the corresponding results were 57.9% (22 of 38) and 66.7% (8 of 12 patients), *p* = 0.589.

## Discussion

Tuberculosis is a global health burden. It has been estimated that there were 10 million cases globally in 2019, with 1.4 million deaths, and that tuberculosis results in more deaths than any other infectious agent [1]. The burden of TB is particularly onerous in low- and middle-income countries, who are faced with high caseloads and limited resources, including a shortage of intensive care beds. Data suggests that delays in initiation of TB treatment are associated with increased mortality [2, 3]. Urinary LAM offers the prospect of rapid point-of-care diagnosis of TB and has been studied in both outpatient and general hospital settings. LAM-based strategies may shorten the time to TB diagnosis and diagnose cases of TB that may have been missed with standard testing methods, with a suggestion that this may improve patient outcomes [13, 17, 19].

The burden of tuberculosis on critical care services is not well described, but is likely to be significant in TB endemic areas, with data from the study ICU indicating that 18% of patients admitted to the ICU with community acquired pneumonia had microbiologically proven TB [20]. Patients with TB requiring ICU admission have a high mortality. Data from an ICU in a TB endemic area reported an ICU mortality of 44.2%, with other data suggesting a mortality of 79% in patients with TB who required mechanical ventilation [21, 22]. Although unproven, rapid diagnosis and treatment of TB may be particularly important in this high-risk group of patients. It is unclear whether urinary TB LAM arises from blood stream antigen spread from remote sites of tuberculosis or from dissemination of *Mycobacterium tuberculosis* to the kidney and subsequent shedding of antigen from sites of infection in the kidney, although the latter hypothesis is increasingly favoured [23]. The improved sensitivity of TB LAM in hospitalised patients versus outpatients; and in HIV positive patients, especially those with low CD4 counts, is likely related to the increased risk of bloodstream spread or dissemination with the abovementioned factors. Our hypothesis was that, irrespective of the mechanism of shedding of TB LAM into the urine, critically ill patients would be more likely to have a higher burden of TB and TB LAM would be more sensitive in critically ill patients, and would be less dependent on HIV status and CD4 count [24]. We thus aimed to assess the diagnostic performance of TB LAM as a rapid bedside diagnostic tool in all adult patients investigated for TB during their ICU admission.

As far as the authors are aware this the first study evaluating the use of urinary LAM in the ICU setting. All patients required mechanical ventilation, with the majority having multiorgan failure. These findings and the high ICU mortality rate of 60% highlight the severity of illness in the cohort. The incidence of TB was high, with 42% of patients investigated for TB having the composite outcome of confirmed or probable TB, and 24% having confirmed TB. As expected, the incidence of HIV was high at 58%.

The sensitivity of urinary LAM for confirmed TB in our study was 50%. This is greater than that seen in outpatients but is broadly similar to that seen in a general hospital population [11]. The finding with respect to outpatients was anticipated, however we expected the sensitivity of urinary LAM to be higher in patients admitted to ICU than in a general cohort of hospitalised patients [24]. This may be due to the fact that there is little difference in TB burden/haematogenous spread between hospitalised patients and ICU patients as in TB endemic areas many patients with advanced tuberculous disease are treated in general wards and are not admitted to ICU. The inclusion of HIV negative patients and patients with high CD4 counts would have been expected to have led to a lower sensitivity, however the severity of illness in our cohort may have counterbalanced this effect.

The specificity of TB LAM in the current study was high, ranging from 84.2 to 93.1% depending on whether the diagnostic category was confirmed TB or the composite outcome of confirmed and probable TB. This is broadly in keeping with the range of specificities reported in different populations by Bjerrum et al. but is lower than figures of close to 99% that have been reported [11, 12]. The interpretation of apparent false positive urinary LAM results is complex. A false positive LAM may also result from cross-reactivity with LAM from infections with non-tuberculous mycobacteria or from bacterial or fungal contamination of the urinary specimen [17, 25]. Ideally urinary LAM testing is conducted on mid-stream urine specimens to reduce the risk of contamination; however, this was not possible in critically ill patients and thus catheter specimens were used in the current study, with a theoretically higher risk of bacterial or fungal contamination of the urine specimen. Alternatively, the apparent false positive LAM results may be true positive results, with standard TB testing being prone to false negative results. A true positive urinary LAM and false negative sputum/endotracheal aspirate GeneXpert may be especially likely in the setting of extrapulmonary tuberculosis. In the current study, however, all patients had either pulmonary tuberculosis or disseminated TB with pulmonary involvement, with no patients having isolated extrapulmonary TB.

There is a well-established association between HIV positivity and low CD4 count and increasing LAM sensitivity [11, 14]. We did not, however, observe an association between HIV status or CD4 count and LAM positivity in this study. This must be interpreted with caution as the study was underpowered to assess such an association and this finding may well be a false negative. Further studies should attempt to further elucidate whether HIV status and CD4 counts are surrogates for disease severity and thus mycobacterial burden or dissemination or are independent determinants of LAM sensitivity. This would have important implications for both ICU and non-ICU settings.

The potential clinical significance of the above findings warrants further discussion. Based on the results of this study, urinary TB LAM does not have adequate sensitivity in ICU patients to be used as a standalone diagnostic test. It cannot rule out TB as it failed to detect 50% of patients with confirmed TB. TB LAM can however detect patients in ICU who are at high risk of having TB faster than other currently available diagnostic modalities. Examining the data for LAM positive patients with confirmed TB is illustrative. In 3 patients TB LAM, GeneXpert and auramine were positive. Assuming a real-world turnaround time of 1–2 days for GeneXpert and auramine, urinary LAM would have allowed for the diagnosis of TB 24–48 h before standard testing. In the patient with a positive LAM and TB diagnosed on histology, LAM would have allowed for diagnosis of TB days or weeks (depending on histopathology availability and turnaround times) before definitive diagnosis. Similarly, in 2 patients with positive TB LAM results, other rapid diagnostic tests were negative, and TB was only confirmed on TB culture, potentially expediting the definitive diagnosis of TB by up to 6 weeks. Urinary TB LAM may thus realistically expedite the diagnosis of TB in ICU patients by days to weeks.

The logical assumption is that earlier diagnosis of TB will result in earlier initiation of antituberculous drugs and improved clinical outcomes. This study did not evaluate the effects of TB LAM on time to initiation of TB treatment and clinical outcomes and thus cannot assist in this regard. Pragmatically, however, the impact of TB LAM on initiation of TB treatment in ICU patients will depend on the treating physician’s approach to empiric TB therapy. For physicians who are likely to initiate empiric TB therapy, TB LAM is unlikely to result in a shorter time to treatment, while with physicians who are unwilling to prescribe empiric therapy, LAM is likely to shorten time to treatment. There is no data on whether earlier initiation of TB treatment improves clinical outcomes in ICU patients. In the absence of such data though it appears reasonable to utilise strategies that would lead to earlier, appropriate, initiation of TB treatment in patients at high risk of adverse clinical outcomes. Furthermore, while not a specific objective of this study, urinary TB LAM may allow for the identification of extrapulmonary disease, where standard rapid diagnostic modalities are less sensitive, or pulmonary disease, in patients where respiratory sampling is unfeasible or inadequate.

The main patient-centred risk of early initiation of TB treatment before confirmatory diagnostic testing is unnecessary exposure to the adverse effects of TB treatment. The reasonable specificity shown in this study, suggests that this would be an uncommon occurrence and thus concerns over possible false positive urinary LAM results should not stop initiation of TB treatment in patients with an appropriate clinical presentation. Given the concerns regarding a possible increased risk of false positive results with catheter-based urine samples, every attempt must be made to ensure the urinary LAM specimen is fresh and uncontaminated. We would advise clamping the urinary catheter and obtaining a specimen from the catheter as soon as possible after clamping and would strongly caution against using a specimen from the urinemeter box or bag.

### Limitations

Limitations to the current study include the relatively small sample size. A consequence of this small sample size is the wide confidence intervals for the indices of LAM diagnostic performance. According to the recommendations of Drain et al. for studies evaluating the performance of non-sputum-based biomarker tests for the diagnosis of TB, sample sizes of 10–40 times greater than the study sample would be required to obtain narrow confidence intervals [26]. Given the limited ICU facilities in TB endemic areas, such large sample sizes are unlikely to be feasible without large multicentre studies over extended timeframes. In the interim this study provides both guidance to physicians treating ICU patients and a basis for future studies. The decision to investigate patients for tuberculosis, was at the discretion of the treating intensivist and this may have resulted in bias in terms of patients included in the study. It should not however have impacted on the study outcome. A significant limitation is one inherent in tuberculosis studies: the lack of a gold standard with adequate sensitivity and specificity for diagnosing TB. To compensate for this the current study has presented the performance of TB LAM relative to two diagnostic categories and has attempted to highlight areas of uncertainty in the results.

## Conclusion

Urinary TB LAM offers the possibility of rapid bedside diagnosis of TB in critically ill patients, and potentially shortens the time to initiation of appropriate TB treatment. Urinary TB LAM should be considered in patients admitted to ICU where TB is likely. A positive TB LAM should prompt consideration of early initiation of TB treatment while the results of further diagnostic testing are awaited. The urinary LAM assay is an affordable and accessible diagnostic tool which could prove valuable in intensive care units in TB endemic areas. Further testing is needed on the possible utility of urinary LAM in ICU.

## Data Availability

Data will be made available to interested parties on reasonable request and with ethics committee approval. The corresponding author (KdV) should be contacted for any data requests.

## Abbreviations

GXP: GeneXpert
ICU: Intensive care unit
LAM: Lipoarabinomannan
TB: Tuberculosis
